# Comparison of cardiovascular risk factors and dietary intakes among Javanese Surinamese and South-Asian Surinamese in the Netherlands. The HELIUS study

**DOI:** 10.1186/s13104-016-2352-4

**Published:** 2017-01-06

**Authors:** Qaisar Raza, Marieke B. Snijder, Jacob C. Seidell, Ron J. G. Peters, Mary Nicolaou

**Affiliations:** 1 Department of Health Sciences, Faculty of Earth and Life Sciences, VU University Amsterdam, De Boelelaan 1085, Kamer O534, 1081 HV Amsterdam, The Netherlands; 2 Department of Public Health, Academic Medical Centre, University of Amsterdam, Amsterdam, The Netherlands; 3 Department of Cardiology, Academic Medical Centre, University of Amsterdam, Amsterdam, The Netherlands

**Keywords:** Cardiovascular disease, Diet, Ethnic differences, South Asian Surinamese, Javanese Surinamese

## Abstract

**Background:**

Ethnic differences regarding the percent of non-communicable diseases have been shown in Asia but the studies on Asian subgroups living in the western countries regarding percent of cardiovascular risk factors and dietary intakes have been scarce. Therefore we compared the percent of cardiovascular risk factors and dietary intakes between Javanese Surinamese who are originally from Indonesia and South-Asian Surinamese who are originally from India.

**Methods:**

Cross-sectional baseline data of the HELIUS (Healthy Life in an Urban Setting) study were used, including data of 2935 Surinamese participants (197 of Javanese and 2738 of South-Asian origin) out of which 1160 participants (78 Javanese and 1082 South-Asian) additionally reported dietary intake data. Descriptive statistics were used to compare the two ethnic groups regarding cardiovascular disease, diabetes, obesity, hypertension and hypercholesterolemia; in addition, dietary intake of foods like vegetables, red meat, fruit, high fibre foods, low fibre foods, high fat and low fat dairy products, chicken and sugar sweetened beverages were also compared between the two groups. Binary logistic regression analyses were used to adjust for age and sex when comparing the two groups.

**Results:**

South-Asian Surinamese had a significantly higher percent of abdominal obesity (OR 2.44; CI 1.66–3.57), cardiovascular disease (OR 2.55; CI 1.48–4.35) and diabetes (OR 2.77; CI 1.67–4.60) as compared with Javanese Surinamese after adjustment for age and sex. The percent of obesity (BMI), hypertension, and lipids was not significantly different between the ethnic groups. Javanese Surinamese had a significantly higher intake of red meat and a significantly lower intake of dairy products as compared with South-Asian Surinamese. Intakes of vegetables, grains, fish, fruits, tea and coffee did not significantly differ between the ethnic groups. Both groups showed intake of considerable amount of sugar sweetened beverages.

**Conclusions:**

Public health practitioners in the Netherlands and elsewhere in the world should take into account the ethnic subgroup differences within the broader groups like Asians when developing interventions related to health among ethnic minorities.

## Background

Cardiovascular disease (CVD) is the leading cause of death among Asians [1]. Previous studies in Asia have shown ethnic differences regarding the percent of cardiovascular disease and related non-communicable diseases between different Asian groups living in Asia [2, 3]. These studies have shown, for instance, that South-Asians (Indians) living in Singapore have a higher percent of coronary heart disease, diabetes and obesity as compared to Malays living in Singapore.

Diet is presumed to be a key lifestyle factor contributing to the increased risk of cardiovascular disease among the Asian population [4, 5]. The traditional Asian diet varies by region. For example, the South-Asian cuisine mostly consists of seasonal fruits and vegetables, meat, rice, legumes, dairy products, fish in the coastal areas, and roti, which is also known as chapatti (Indian Subcontinent flat bread, made from stoneground wholemeal flour, traditionally known as ‘Atta’ flour). In addition, most of the South Asians practicing Hindu religion avoid beef and may be vegetarian, while Muslim groups (including Pakistanis and Bangladeshis) avoid pork. A major portion of the Southeast Asian cuisine consists of rice, fish, vegetables, poultry and coconut. Southeast Asia consists of Muslim countries such as Indonesia and Malaysia, Buddhist countries like Cambodia, Laos and Thailand and Christian countries like the Philippines.

Certain dietary items have a protective effect against cardiovascular disease, diabetes and obesity such as fruits, vegetables, fibrous foods and fish [6, 7]. On the other hand there are dietary items which increase the risk such as excessive use of sugar, fat and carbohydrates through excessive consumption of soft drinks, milk based products, meat and rice [8–11]. There is evidence in favor of high fat dairy products as compared to low fat dairy products in keeping the weight down and thus eventually reducing the risk of heart disease [12, 13].

Previous studies among Asian migrants living in western countries have pointed towards disease and diet differences among different Asian ethnicities [14, 15]. However, heterogeneity regarding the percent of non-communicable diseases and intake of dietary items among Asians living in western countries has not been adequately studied. The Netherlands hosts many ethnic groups including Surinamese of Asian origin who originally migrated as labourers from regions of Indonesia (Java) and South Asia (North India) to Suriname and then to the Netherlands. Previous studies have shown that South-Asian Surinamese have a higher percent of CVD and related risk factors as compared to the Dutch host population [16, 17]. South-Asian Surinamese follow their traditional dietary pattern consisting of rice/noodles and chicken [18]. To our knowledge there has been no study exploring the percent of non-communicable diseases and intake of dietary items among the Javanese Surinamese.

South-Asian Surinamese and Javanese Surinamese originate from different countries, thus carrying different genetic and cultural backgrounds. At the same time these two ethnicities lived together in Surinam before migrating to the Netherlands. After the independence of Suriname in 1975, a large wave of Surinamese settled in Amsterdam. The present study addresses ethnic differences regarding the percent of non-communicable diseases and intake of dietary items between immigrants from the same country but with different cultural and genetic backgrounds. This information can help in developing tailored and culturally sensitive interventions to tackle non-communicable diseases. Therefore, the aim of this study is to compare the percent of cardiovascular risk factors and dietary intakes between two Asian groups, namely Javanese Surinamese and South-Asian Surinamese, living in Amsterdam, the Netherlands.

Thus we will answer following two research questions in this study.

1. What are the differences regarding percent of cardiovascular disease and related risk factors listed below between South Asian Surinamese and Javanese Surinamese?
  Obesity
  Diabetes
  Hypertension
  Cholesterol levels
2. What are the differences in the intake of food items related to cardiovascular disease and related risk factors listed below between South Asian Surinamese and Javanese Surinamese?
  Vegetables
  Fruits
  Red meat
  Fibrous foods
  Dairy
  Sugar sweetened beverages

## Methods

### Study design and sample

Cross-sectional baseline data of the HELIUS (Healthy Life in an Urban Setting) study were used. The HELIUS study is a cohort study among the six largest ethnic groups living in Amsterdam, the Netherlands, and baseline data collection started in January 2011. The overall aim of the HELIUS study is to examine the health disparities across different ethnic groups and its causes. The population, aged 18–70 years, was randomly sampled from the Amsterdam municipality register, stratified by ethnicity (based on county of birth, and country of birth of the parents). More details on the HELIUS study design have been published elsewhere [19]. For the current study, data collected between January 2011 and June 2015 were used. All participants of Asian origin, namely Javanese Surinamese (n = 197) and South-Asian Surinamese (n = 2738), were selected. Of those, 78 Javanese Surinamese and 1082 South-Asian Surinamese also had extensive dietary intake data.

### Measurements

Anthropometric measures were performed using standardised protocols [19]. BMI was calculated as weight (kg) divided by height (m) squared. Overweight was defined as BMI ≥ 23 kg/m^2^ and <27 kg/m^2^, and obesity as BMI ≥ 27 kg/m^2^ based on appropriate cut-off points for Asians [23]. Waist circumference (WC) and waist-to-hip ratio [WHR calculated by dividing waist circumference (cm) by hip circumference (cm)] were measured and overweight/obesity was defined as WC > 90 cm and >80 cm, and WHR > 0.90 and >0.80, in men and women, respectively, based on appropriate cut-off points for Asians as described by world health organization (WHO) [24].

Prevalent cardiovascular disease (CVD) was defined as the presence of intermittent claudication, possible infarction, and/or angina pectoris according to the Rose questionnaire [25]. Diabetes was defined on the basis of self–report, elevated fasting glucose (≥7 mmol/l), increased HbA1c (≥48 mmol/mol) and/or the use of glucose lowering medication. High blood pressure was defined as self-reported hypertension, systolic blood pressure (SBP) ≥140 mmHg, diastolic blood pressure (DBP) ≥90 mmHg, and/or the use of BP lowering medication. Hypercholesterolemia was defined as total cholesterol level >5.2 mmol/l. High LDL was defined as LDL level >3.4 mmol/l. Low HDL was defined as HDL <1 mmol/l for men and <1.3 mmol/l for women. High triglycerides was defined as triglycerides >2.0 mmol/l [26].

### Measurements for dietary intake variables

Food intake was measured using ethnic specific FFQs that were designed for the HELIUS study [27]. The FFQs with approximately 200 food items were used to collect information about the frequency and the amount of intake of the respective food items in the previous 4 weeks. In the current study we used information about intake of foods presumably relevant for cardiovascular disease. These foods included vegetables, refined grains including rice and noodles, high fibre products, fish, red meat, chicken, fruit, fruit juice, sugar sweetened beverages, high fat dairy products, low fat dairy products, tea and coffee [6, 7, 9, 10].

The food groups were combined as follows: ‘Vegetables’ consists of all fresh, frozen, canned vegetables not including legumes or potatoes; ‘fish’ comprises of fatty and lean fish, crustaceans and molluscs and ‘red meat’ includes all red meat including organ and minced meat. Low fibre and refined grains consists of all low fibre bread, crackers, breakfast cereals, pasta, rice, noodles, pancakes; high fibre products and wholegrain cereals consists of all high fibre bread, crackers, Breakfast cereals, pasta, Bulgur and rice; sugar sweetened beverage comprises of sweetened fruit drinks, cordials, soft drinks, energy and sport drinks, ice lollies; high fat dairy products group is made up of high fat milk, milk-based puddings, yoghurt, ice cream, cream, cheese; low fat dairy products group comprises of reduced and fat free milk, yoghurt, puddings and cheese; fruit consists of all fresh, frozen or canned fruits; fruit juices consist of unsweetened and fresh juices.

### Statistical analysis

Descriptive statistics were used to calculate means and percentages for demographic variables such as age, sex, marital status, educational level, gender and disease variables like CVD, diabetes, hypertension and hypercholesterolemia. Dietary variables were not normally distributed, therefore we reported medians and inter quartile ranges. We tested for statistical significant differences in dietary intakes between Javanese Surinamese and South-Asian Surinamese using Mann–Whitney test. Furthermore, binary logistic regression analyses were used to analyse the differences in disease percent between South-Asian Surinamese and Javanese Surinamese adjusted for age and sex. Data were analysed using SPSS, version 21.

## Results

### Sociodemographic characteristics

Table 1 shows the demographic characteristics of the Javanese Surinamese and South-Asian Surinamese study population. The mean age of both Asian groups was ±45 years. There were relatively more women in the Javanese Surinamese group as compared with the South-Asian Surinamese. There were more first generation among Javanese Surinamese participants as compared with South-Asian Surinamese, although there were substantially more first generation participants than second generation participants in both ethnic groups. In terms of marital status, a larger percentage of South-Asian Surinamese were married as compared with Javanese Surinamese. A larger percentage of South-Asian Surinamese reported having a university degree as compared to Javanese Surinamese.

**Table 1:** Sociodemographic characteristics of Javanese Surinamese and South-Asian Surinamese participants

	Javanese Surinamese (n = 197)	South-Asian Surinamese (n = 2738)
Age (years)	45.9 (11.7)	45.4 (13.3)
Men (%)	39.6	45.1
1st generation (%)	87.3	76.7
Marital status (%)		
Married/registered partnership	29.4	34.5
Living together	21.4	10.1
Unmarried, have never been married	38.1	32.9
Divorced or separated	11.2	22.4
Educational level (%)		
Never been to school or elementary schooling	10.3	15.4
Lower vocational schooling or lower secondary schooling	34.4	33.4
Intermediate vocational schooling or intermediate/higher secondary education schooling	35.9	28.9
Higher vocational schooling or university	19.5	22.3

### Cardiovascular risk factors

Although mean BMI or general overweight/obesity did not differ between the two groups (Table 2), South-Asian Surinamese had a significantly higher percent of central obesity based on WC and WHR as compared to Javanese Surinamese (Table 3). This was consistent for both men and women, although associations in women were weaker. South Asian Surinamese also had a significantly higher percent of self-reported CVD, diabetes and low HDL as compared with Javanese Surinamese, after adjustment for age and sex (Table 3). Other lipids, and the percent of hypertension, did not significantly differ between the ethnic groups.

**Table 2:** Mean (SD) anthropometric measurements and blood lipid levels among Javanese Surinamese and South-Asian Surinamese participants

	Javanese Surinamese (n = 197)	South-Asian Surinamese (n = 2738)
BMI Overweight (BMI ≥ 23 kg/m^2^) Obesity (BMI ≥ 27 kg/m^2^)	25.8 (4.5)	26.2 (4.7)
WC (men) Overweight/obesity (WC > 90 cm)	89.9 (11.2) **	93.7 (12.0)
WHR (men) Overweight/obesity (WHR > 0.90)	0.93 (0.06) **	0.97 (0.07)
WC (women) Overweight/obesity (WC > 80 cm)	85.4 (11.4)	89.9 (13.2)
WHR (women) Overweight/obesity (WHR > 0.80)	0.86 (0.07)	0.93 (0.06)
Total cholesterol Hypercholesterolemia (total cholesterol level >5.2 mmol/l)	5.1 (1.0)	4.97 (1.03)
LDL High LDL (LDL level > 3.4 mmol/l)	3.1 (0.92)	3.1 (0.93)
HDL Low HDL (HDL < 1.1 mmol/L)	1.4 (0.39)	1.3 (0.37)
Triglycerides High triglycerides (triglycerides >2.0 mmol/l)	1.2 (1.1)	1.1 (0.82)

* P < 0.05, ** P < 0.01 indicates statistically different from South-Asian Surinamese

**Table 3:** Percent and odds ratio of cardiovascular disease and related risk factors among Javanese Surinamese and South-Asian Surinamese participants

	Javanese Surinamese (n = 197)	South-Asian Surinamese (n = 2738)	Differences between ethnic groups Adjusted for age and sex: Javanese Surinamese were taken as a reference group	
			OR	95% CI
Overweight based on BMI (%)	39.1	38.4	0.95	0.70–1.27
Obesity based on BMI (%)	32.5	37.7	1.31	0.95–1.78
Obesity based on high waist circumference (%)	56.9**	70.1	2.20	1.60–3.02
Obesity based on high waist-to-hip Ratio (%)	74.6**	83.8	2.44	1.66–3.57
CVD according to Rose	7.7**	17.2	2.55	1.48–4.35
Diabetes	8.2**	18.7	2.77	1.67–4.60
Hypertension	37.8	42.4	1.28	0.91–1.79
High total cholesterol	41.1	39.5	0.94	0.70–1.26
High LDL	32.8	36.9	1.18	0.86–1.62
Low HDL	26.9**	41.2	1.89	1.34–2.65
High triglycerides	13.7	9.6	0.64	0.41–0.99

* P < 0.05, ** P < 0.01 indicates statistically different from South-Asian Surinamese

### Dietary intakes among Javanese Surinamese and South-Asian Surinamese

Javanese Surinamese reported a significantly higher intake of red meat as compared to South-Asian Surinamese (Table 4). South-Asian Surinamese reported a significantly higher intake of milk based products (both high-fat and low-fat) as compared to Javanese Surinamese. Both groups reported using greater amounts of low-fat dairy products as compared with high-fat dairy products. Javanese Surinamese reported a median intake of vegetables of 107.7 g per day and a median intake of chicken of 35.7 g per day while South-Asian Surinamese reported a median intake of vegetables of 93.4 g per day and a median intake of chicken of 34.8 g per day. There was a high daily intake of sugar sweetened beverages by both ethnic groups.

**Table 4:** Food intakes (grams per day) among Javanese Surinamese and South-Asian Surinamese participants

Food groups	Javanese Surinamese (n = 78)	South-Asian Surinamese (n = 1082)	P values
Vegetables	107.7 (58.5–190.0)	93.4 (37.5–165.2)	0.43
Low fibre and refined grains	164.0 (75.3–251.4)	167.3 (97.9–251.7)	0.63
High fibre products and wholegrain cereals	81.4 (51.2–126.2)	83.9 (48.7–140.0)	0.42
Fish	8.9 (3.0–25.1)	8.5 (0.8–17.1)	0.30
Red meat	52.2 (20.0–80.4)**	21.9 (6.2–46.3)	0.00
Chicken	34.8 (14.6–53.5)	35.7 (17.8–57.6)	0.41
Fruit	133.3 (66.4–271.8)	148.8 (63.3–259.0)	0.66
Fruit juice	25.0 (0.0–50.0)	25.00 (0.00–50.0)	
Sugar sweetened beverages	55.4 (2.6–274.8)	52.5 (7.1–200.0)	0.22
High fat dairy products	13.5 (2.7–52.1) *	18.5 (3.4–48.2)	0.04
Low fat dairy products	19.4 (2.2–71.6) **	33.9 (7.0–114.9)	0.00
Tea	352.1 (170.0–680.0)	242.8 (72.8–510.0)	0.06
Coffee	140.0 (9.3–315.0)	105.0 (1.2–280.0)	0.07

Data are medians (25–75 percentiles)

* P < 0.05, ** P < 0.01 indicates statistically different from South-Asian Surinamese

## Discussion

Our study found that South-Asian Surinamese had a significantly higher percent of central obesity, self-reported cardiovascular disease and diabetes as compared to Javanese Surinamese after adjustment for age and sex. South-Asian Surinamese also had a significantly lower intake of red meat and significantly higher intake of dairy products as compared to Javanese Surinamese. No significant differences were observed in the percent of general obesity, hypertension, lipid profile and the intake of other dietary variables.

Studies from Asia have found that different ethnic groups within Asia like Indians and Indonesians have differences regarding the prevalence of cardiovascular risk factors [28–30]. In India 8% of the population is diagnosed with type 2 diabetes [32] whereas in Indonesia diabetes percent was 4.6% in 2010, and expected to rise to 6% by 2030 [35]. In Suriname, South Asian Surinamese have a higher percent of diabetes than other population groups [32]. Thus it seems that people of South Asian origin seem to have higher percent of diabetes in comparison with other ethnicities, irrespective of the place where they live [31, 32]. Our findings are consistent with this observation; Javanese Surinamese who originate from Indonesia (Java) and South-Asian Surinamese who originate from India also differed regarding the percent of CVD related risk factors. Consistent with the literature, South Asian Surinamese had higher levels of central obesity and diabetes than Javanese Surinamese. Nonetheless, both groups in our study had higher rates of diabetes than their counterparts in India and Indonesia; 18.7 and 8.2% for the South Asian and Javanese Surinamese respectively, implying higher risk in migrants, potentially due to migration-related factors. A study among Turkish, Moroccan and Surinamese regarding the prevalence of obesity in the Netherlands has shown that among second generation migrant men, Surinamese have higher prevalence of overweight as compared to Moroccans and Turkish while the overweight levels for women of first generation varied from 36% among Surinamese to 50% among Moroccans [46].

Javanese Surinamese and South-Asian Surinamese originally migrated from the region of Southeast Asia and South Asia, respectively, to Surinam and then to the Netherlands. Southeast Asia comprises of countries like Philippines, Malaysia, East Timor, Indonesia, Brunei, and Singapore whereas South Asia comprises of countries like India, Pakistan, Bangladesh, Sri Lanka and Nepal. Javanese Surinamese were brought from the Java region in Indonesia and South-Asian Surinamese were brought from the modern day Indian states of Bihar, Utter Pradesh and adjoining areas by Dutch to work on sugar plantations in Surinam in late 18th and early nineteenth century. The mass migration of Surinamese to the Netherlands occurred after 1975, when Surinam announced independence from the Netherlands; more than hundred thousand Surinamese migrated to the Netherlands at that time. Most of these Surinamese settled around big cities in the Netherlands including Amsterdam, Rotterdam, Utrecht and Den Haag. There are 148,443 South Asian Surinamese and 73,975 Javanese Surinamese living in Surinam and there are 151,000 South Asian Surinamese and 22,000 Javanese Surinamese living in the Netherlands [20–22].

The differences in CVD risk factors and dietary intakes between South Asian Surinamese and Javanese Surinamese, however, cannot be explained by their migration history to and from Suriname or by socio-economic factors. Both ethnic groups migrated to Suriname as contract workers in the late nineteenth century to fill labour gaps after the abolition of slavery. Subsequent migration to the Netherlands followed a similar trajectory for both groups; voluntary migration after the independence of Suriname. In the Netherlands, the groups have had the advantage of speaking the Dutch language. Currently there are 350,000 Surinamese living in the Netherlands, amongst whom first generation migrants generally have low levels of education and high unemployment rates [33–35], with few differences between the South Asian and Javanese Surinamese [36].

We studied the intake of some key dietary indicators and the results showed differences between the two ethnic groups. South-Asian Surinamese reported lower intake of vegetables and higher intake of dairy products (both high and low fat varieties) as compared to Javanese Surinamese; though both groups were using higher amounts of low fat dairy products as compared to high fat dairy products. Lower intake of vegetables is related to the increased risk of stroke [37]. Excessive consumption of dairy products could lead to weight gain although recently there is evidence in favor of high fat dairy products as compared to low fat dairy products in keeping the weight low [12, 13]. Our study also found that Javanese Surinamese had a significantly higher intake of red meat as compared to the South-Asian Surinamese. Red meat consumption is associated with increased cardiovascular disease mortality [38].

Previous studies of the diet of Surinamese origin migrants in the Netherlands have included South Asian and African Surinamese and have shown that these groups have similar dietary habits and fidelity to a typical Surinamese dietary pattern characterized by intakes of rice, noodles, chicken and fish [39]. However, despite these similarities, the South Asian group had lower intakes of meat and meat products and higher intakes of dairy products compared to African Surinamese and consistent with what was found in this study. Studies of similar ethnic groups in different settings also show some similarity with our findings. Asian Indian immigrants living in United States have also shown lower intake of vegetables and more intake of dairy than the recommended amounts; though there were variations within the Asian Indian group regarding the intake of dietary items [40]. An earlier study has shown that Southeast Asian immigrants have a higher intake of red meat as compared to South Asian immigrants living in New Zealand [41]. The generalizability of our results might not be possible to South Asian and Javanese Surinamese populations living as immigrants in other western countries due to different food environments but these comparisons between Asian groups living in different western countries may be illustrative of the consistency of traditional dietary habits, despite differing migration trajectories. The lower consumption of meat among South Asian Surinamese in our study and also elsewhere may be related to the religious background of the South Asian Surinamese of whom the majority (65%) is reported to be Hindus [42]. Nonetheless, a shared environment does also play a role; we were not able to find any significant differences between the two ethnic groups for the intake of other dietary items like refined grains, high fiber products, fish, chicken, fruit, tea and coffee. Both ethnic groups reported a relatively high intake of sugar sweetened beverages and low fibre and refined grains. Thus, at this level, the results of our study seem to be in line with a review of the change in dietary habits of immigrants after migration to the European countries which has shown that migrants tend to shift towards western dietary lifestyle including consuming more sugar sweetened beverages and low fiber foods which results in increasing prevalence of cardiovascular risk factors [43]. These results show unhealthy changes in the diet of immigrants after migration to western countries which is a concern for public health professions and could be addressed through tailored lifestyle interventions.

The findings of our study need to be taken in light of several limitations. The number of Javanese Surinamese was relatively small, reflecting their relatively small representation within the Surinamese population in the Netherlands where approximately 40–45% are of South Asian origin and 10–14% Javanese [44, 45] out of total Surinamese population of 350,000 [33]. Thus, in general we had an appropriate sample size for both South Asian and Javanese Surinamese but we had a rather small sample size for dietary intake of Javanese Surinamese, so we should be careful in generalizing these results to the entire Javanese Surinamese population in the Netherlands. In addition, our study had a cross sectional design and is descriptive in nature. Our study provides an explorative picture of differences in diet and health of the two Asian groups living in the Netherlands but it does not account for causality regarding the diet related diseases.

## Conclusions

Our study found differences regarding the percent of cardiovascular risk factors and dietary intake between Javanese Surinamese and South-Asian Surinamese. South Asian-Surinamese had a significantly higher percent of abdominal obesity, self-reported CVD and diabetes, but did not differ in general obesity, hypertension and lipid profile. Both groups had a high percent of all cardiovascular related risk factors. Lower intake of vegetables and higher intake of dairy products among South Asian Surinamese as compared to Javanese Surinamese is a potentially unhealthy trend. In addition, higher intakes of red meat among Javanese Surinamese as compared to South Asian Surinamese should probably be regarded as unhealthy trend. The intake of low fibre refined grains and sugar sweetened beverages among both ethnicities could lead to an increased risk for the development of diabetes and other cardiovascular risk factors. CVD risks could be reduced by encouraging the intake of vegetables among South-Asian Surinamese and reducing the intake of red meat among Javanese Surinamese. In addition, the intake of low fibre refined grains should be reduced and replaced with high fibre whole grain foods. Similarly sugar sweetened beverages should be replaced with water among both groups.

## Abbreviations

CVD: cardiovascular disease
WC: waist circumference
WHR: waist hip ratio
